# Evaluation of Viral Transport Medium and Inactivation Transport Medium Under Different Storage Conditions for the Detection of SARS-CoV-2 by Real-Time Polymerase Chain Reaction Using the cobas® 8800 System

**DOI:** 10.7759/cureus.113130

**Published:** 2026-07-21

**Authors:** Kamini S Ranawat, Lalita Verma, Nita Pal, Saroj Hooja, Bharti Malhotra

**Affiliations:** 1 Microbiology, SMS Medical College, Jaipur, IND

**Keywords:** cobas® 8800 system, covid-19, inactivation transport medium, rt-qpcr sars-cov-2, viral transport medium

## Abstract

Background: Accurate and timely diagnosis is vital for the surveillance and containment of COVID-19. Traditional viral transport medium (VTM) for sample collection requires strict cold chain maintenance, which poses biosafety and logistical challenges, especially in resource-limited or remote settings. Inactivation transport medium (ITM), containing guanidinium thiocyanate, is designed for safe, room temperature (RT) transport. This study compared ITM with VTM for RNA stability and diagnostic efficiency using the cobas® 8800 system.

Methods: In this comparative observational study, oropharyngeal swabs were collected from 80 SARS-CoV-2-positive patients and 20 healthy controls. For each participant, three swabs were collected: one for ITM and two for VTM. Samples in ITM were stored at RT (25-32 °C), and those in VTM at 4 °C and RT. All samples were tested by reverse transcription quantitative polymerase chain reaction (RT-qPCR) (cobas® 8800) on days zero, one, two, and three. Cycle threshold (Ct) values for ORF1a/b and E genes were compared across groups to assess RNA stability and detection efficiency.

Results: Ct values remained stable across ITM and VTM at 4 °C for up to 72 hours, while VTM at RT showed a trend of higher Ct values, particularly by day three. Although not statistically significant, degradation was more evident in VTM at RT. A significant difference was observed for the E gene on day one (p = 0.001), favouring ITM. Negative controls remained consistently negative across all media and temperatures.

Conclusions: ITM provides RNA preservation and diagnostic performance for SARS-CoV-2 detection comparable to VTM, with the added benefits of RT stability and enhanced biosafety. Use of ITM may eliminate the need for cold chain logistics and reduce biohazard risks, improving the reliability and flexibility of COVID-19 testing in varied settings.

## Introduction

Severe acute respiratory syndrome coronavirus 2 (SARS-CoV-2) is a novel virus of the Coronaviridae family and the etiological agent of coronavirus disease 2019 (COVID-19), responsible for the global pandemic [1]. The first case in India was reported in the state of Kerala on January 30, 2020 [2]. As of 1 April 2026, more than 45 million cases of COVID-19 infection have been reported in India [3]. Periodic surges are still being reported due to mutations in the virus, which continue to produce new subvariants. To protect the high-risk groups and control the virus as a persistent endemic threat, accurate and timely diagnostic testing remains essential for disease surveillance, clinical management, and containment of the COVID-19 pandemic. In view of the emergence of SARS-CoV-2 variants, samples have to be tested quickly for timely results. The Centers for Disease Control and Prevention (CDC) recommends the collection of oropharyngeal (OP)/nasopharyngeal (NP) specimens in a viral transport medium (VTM) or saline, followed by RNA extraction and reverse transcriptase polymerase chain reaction (RT-PCR) for testing the presence of SARS-CoV-2 in patient samples [4,5]. Real-time reverse transcription-quantitative polymerase chain reaction (RT-qPCR) is regarded as the gold standard for the diagnosis of COVID-19 from NP and/or OP swab specimens collected in VTM [6]. VTM is composed of a balanced salt solution, fetal bovine serum, antifungals, and antibiotics.

Transport in VTM requires the specimen to be kept at 4 °C in order to prevent degradation of viral nucleic acid. As the virus is kept in its infectious state, the specimens pose a significant biohazard both during transport and in the laboratory and require special safety procedures of packaging and transport, which cause a considerable bottleneck in the processing workflow [7]. There is a risk of leakage of clinical samples during transport to a testing laboratory, presenting a biohazard along the transport chain. Additionally, this transportation of samples may be a challenge when collected from remote areas due to limitations in maintaining the cold chain because of power failure or lack of cold chain infrastructure. This can present logistical challenges in the testing process. Rapid inactivation of SARS-CoV-2 is crucial to ensure operator safety during high-throughput testing of clinical samples.

A variety of transport media and lysis buffers containing guanidium hydrochloride and guanidinium thiocyanate have been shown to effectively inactivate the virus while preserving stability of the released RNA [8-11].

Guanidine thiocyanate-based media reportedly inactivate SARS-CoV-2 and are compatible with RT-qPCR assays [12]. GeneTM and eNAT™ (Copan, Brescia, Italy) are two guanidine thiocyanate-based media that have been found to be suitable for collecting OP and NP specimens and help nucleic acids remain stable for long periods [13,14]. VTM is suitable for virus culture, as well as molecular diagnosis, but in circumstances where only molecular diagnosis is required, a transport media not require a cold chain, inactivates the virus, and keeps the nucleic acid intact is desirable. The present study hypothesized the collection of SARS-CoV-2 clinical samples into an inactivation transport medium (ITM) that inactivates the virus and preserves viral RNA at room temperature (RT). ITM is an Indian Council of Medical Research (ICMR) approved medium that contains chaotropic agents, guanidine thiocyanate, and ethanol, and a chelating agent (ethylenediamine tetraacetic acid) that allows transport of specimens without the need for a cold chain and improves the biosafety profile of the entire diagnostic workflow. ITM is a licensed specimen collection medium and swab validated by ICMR, supplied by Promea Therapeutics, Hyderabad, Telangana, India (Licence Number: MFG/ IVD/2020/000122). This study aims to compare the sensitivity of VTM and ITM for SARS-CoV-2 detection and to evaluate RNA stability in specimens stored in these media at 24, 48, and 72 hours using the real-time RT-PCR by the cobas® 8800 system (Roche Molecular Systems, Inc., Branchburg, NJ).

## Materials and methods

This was a comparative observational study conducted in the Department of Microbiology, SMS Medical College, Jaipur, Rajasthan, India, over a period of two months (September-October 2022). The study included adult symptomatic patients admitted to the COVID inpatient (IPD). Patients less than 18 years of age and those who did not give consent were excluded from the study.

A sample size of 80 was calculated at 95.0% confidence interval to verify the expected minimum difference of 2.1 ± 4.2 in the mean cycle threshold (Ct) value of Day 0 in VTM at 4 °C, keeping the study power to 80.0%. The Online Statulator 2023 software was used for sample size calculation.

After approval from the Institutional Research Review Board and Ethical Committee (Approval No: 550 MC/EC/2022 dated 5/8/2022), samples were randomly collected from 80 patients who were reported positive for SARS-CoV-2 by RT-qPCR by routine testing in our laboratory and were admitted to our hospital. In addition, samples were collected from 20 healthy adult volunteers, recruited as negative controls.

After counseling the patient and taking informed consent, a set of three OP samples was collected, one for ITM and the other two for VTM (Vitromed Healthcare, Biotech Park, Jaipur, Rajasthan, India) for this study. A total of 300 samples were collected prospectively; these included 240 samples from 80 SARS-CoV-2-positive patients (two for VTM and one for ITM) and 60 samples from 20 healthy volunteers (two for VTM and one for ITM) that were recruited as negative controls. Samples were transported to the laboratory within one hour of collection. The RT-qPCR test was performed for samples from both the transport media as soon as received in the laboratory on cobas® 8800 (Day 0) to attain an initial Ct value.

The samples in ITM were stored at RT (25-32 °C). One set of samples in VTM was stored at 4 °C in a refrigerator, while the other set of samples in VTM was stored at RT. These samples were processed for RT-qPCR after 24, 48, and 72 hours. Samples from each set of media were processed by different researchers, and the findings were shared with the analyst independently, without disclosing the duration and storage conditions, using codes. Ct values of all the samples were noted and analysed.

RT-qPCR testing on the cobas® 8800 system

All molecular detection of SARS-CoV-2 was performed on the cobas® 8800 system (Roche Molecular Systems, Branchburg, NJ), using the cobas® SARS-CoV-2 assay authorized under Emergency Use Authorization (EUA). This platform is a fully automated, sample-to-result, high-throughput RT-PCR system that integrates specimen preparation, nucleic acid extraction, amplification, and detection within a closed workflow, thereby minimizing contamination risks. All the samples were processed in a Class II biological safety cabinet (BSC II) before being loaded on the cobas® 8800 system.

For testing of the samples, 600 μL of VTM and ITM aliquots equilibrated to RT were transferred into barcoded secondary tubes and loaded on the cobas®8800 system. External controls (positive and negative) were processed in the same way with each run. Amplification was carried out using target-specific primers and fluorescent hydrolysis probes directed against two regions: ORF1 a/b gene, specific to SARS-CoV-2, and E gene, which is conserved across Sarbecoviruses.

The assay also includes an RNA internal control (RNA IC), which was co-extracted and amplified with each clinical sample to monitor both extraction efficiency and PCR inhibition. RT-qPCR and fluorescence-based detection were performed within sealed cartridges. Amplification curves were automatically analysed by the system software, and results were reported as "Detected", "Not Detected", or "Invalid" depending on amplification profiles and control outcomes. According to the manufacturer's instructions, a tested sample was considered SARS-CoV-2-positive if cobas® 8800 showed a Ct value of the open reading frame (ORF) gene with or without the E gene of ≤35.

Statistical analysis

Continuous data were expressed as mean ± standard deviation. Change in Ct value from baseline was calculated to assess RNA stability over time. Since samples from the same patients were tested repeatedly, repeated-measures ANOVA was used for comparison across transport media and time points. Pairwise comparisons were done using a paired t-test with Bonferroni correction. P value < 0.05 was considered statistically significant.

## Results

A total of 80 SARS-CoV-2-positive paired samples were included in the analysis. At baseline, based on VTM E-gene Ct values, 24 (30.00%) samples had Ct values <25, 47 (58.75%) had Ct values between 25 and 30, and 9 (11.25%) had Ct values >30. All positive samples remained detectable in all three storage conditions up to 72 hours.

Mean Ct values increased gradually with time in all groups (Table 1). The increase was the highest in VTM stored at RT. At 72 hours, the mean ORF1a/b Ct value was 28.06 ± 3.36 in VTM at RT, 26.79 ± 3.87 in VTM at 4 °C, and 26.71 ± 3.72 in ITM at RT. Similarly, the mean E-gene Ct value at 72 hours was 29.03 ± 3.44 in VTM at RT, 27.50 ± 3.63 in VTM at 4 °C, and 28.68 ± 4.42 in ITM at RT.

**Table 1:** Mean Ct values of ORF1a/b and E genes in different transport media over 72 hours *Data are presented as mean ± SD. Each group included 80 paired positive samples. Ct = cycle threshold; VTM = viral transport medium; ITM = inactivation transport medium

Gene	Time	VTM at 4 °C, Mean ± SD* (n=80)	VTM at room temperature, Mean ± SD* (n=80)	ITM at room temperature, Mean ± SD* (n=80)
ORF1a/b	Day 0	25.85 ± 4.24	25.85 ± 4.24	25.21 ± 4.02
	Day 1	25.90 ± 4.08	26.16 ± 4.04	25.14 ± 4.02
	Day 2	26.20 ± 4.05	26.95 ± 3.81	25.73 ± 3.91
	Day 3	26.79 ± 3.87	28.06 ± 3.36	26.71 ± 3.72
E gene	Day 0	26.63 ± 4.09	26.63 ± 4.09	26.28 ± 4.16
	Day 1	26.63 ± 4.06	27.03 ± 4.06	25.81 ± 4.29
	Day 2	26.90 ± 4.14	27.56 ± 3.91	26.41 ± 4.20
	Day 3	27.50 ± 3.63	29.03 ± 3.44	28.68 ± 4.42

To assess RNA stability, the change in Ct value from baseline was calculated. A higher rise in Ct value indicates a greater fall in detectable viral RNA. Repeated-measures analysis showed that Ct-value change was significantly affected by transport media, time, and their combined effect for both ORF1a/b and E genes (all p < 0.001) (Table 2).

**Table 2:** Repeated-measures analysis of changes in Ct values from baseline *Repeated-measures ANOVA was used because samples from the same patients were tested repeatedly under different storage conditions and at different time points.

Gene	Factor studied	F value	df	P value
ORF1a/b	Storage condition	16.09	2,158	<0.001
	Time	92.89	2,158	<0.001
	Storage condition × time	10.93	4,316	<0.00
E gene	Storage condition	21.44	2,158	<0.001
	Time	184.93	2,158	<0.001
	Storage condition × time	26.98	4,316	<0.001

Pairwise comparison at 72 hours showed that VTM stored at RT had a significantly higher rise in ORF1a/b Ct value than ITM stored at RT and VTM stored at 4 °C (Table 3). For the E gene, VTM stored at RT had a significantly higher rise in Ct value than VTM stored at 4 °C, while the difference between VTM at RT and ITM at RT was not statistically significant. These findings show that RT storage of VTM was associated with a greater Ct-value rise, especially for the ORF1a/b gene.

**Table 3:** Pairwise comparison of changes in Ct values at 72 hours *Mean difference represents the difference in the change in Ct values from baseline. Paired t-test with Bonferroni correction was used. VTM RT: viral transport medium at room temperature; ITM RT: inactivation transport medium at room temperature

Gene	Comparison	Mean difference*	95% CI	t value	df	Adjusted P value
ORF1a/b	VTM RT vs ITM RT	0.71	0.24 to 1.18	3.01	79	0.021
	VTM RT vs VTM 4 °C	1.28	0.96 to 1.59	8.07	79	<0.001
	VTM 4 °C vs ITM RT	−0.56	−0.95 to −0.17	−2.86	79	0.032
E gene	VTM RT vs ITM RT	0.00	−0.55 to 0.55	0.00	79	1.000
	VTM RT vs VTM 4 °C	1.53	1.23 to 1.82	10.32	79	<0.001
	VTM 4 °C vs ITM RT	−1.53	−2.01 to −1.04	−6.24	79	<0.001

**Figure 1:**
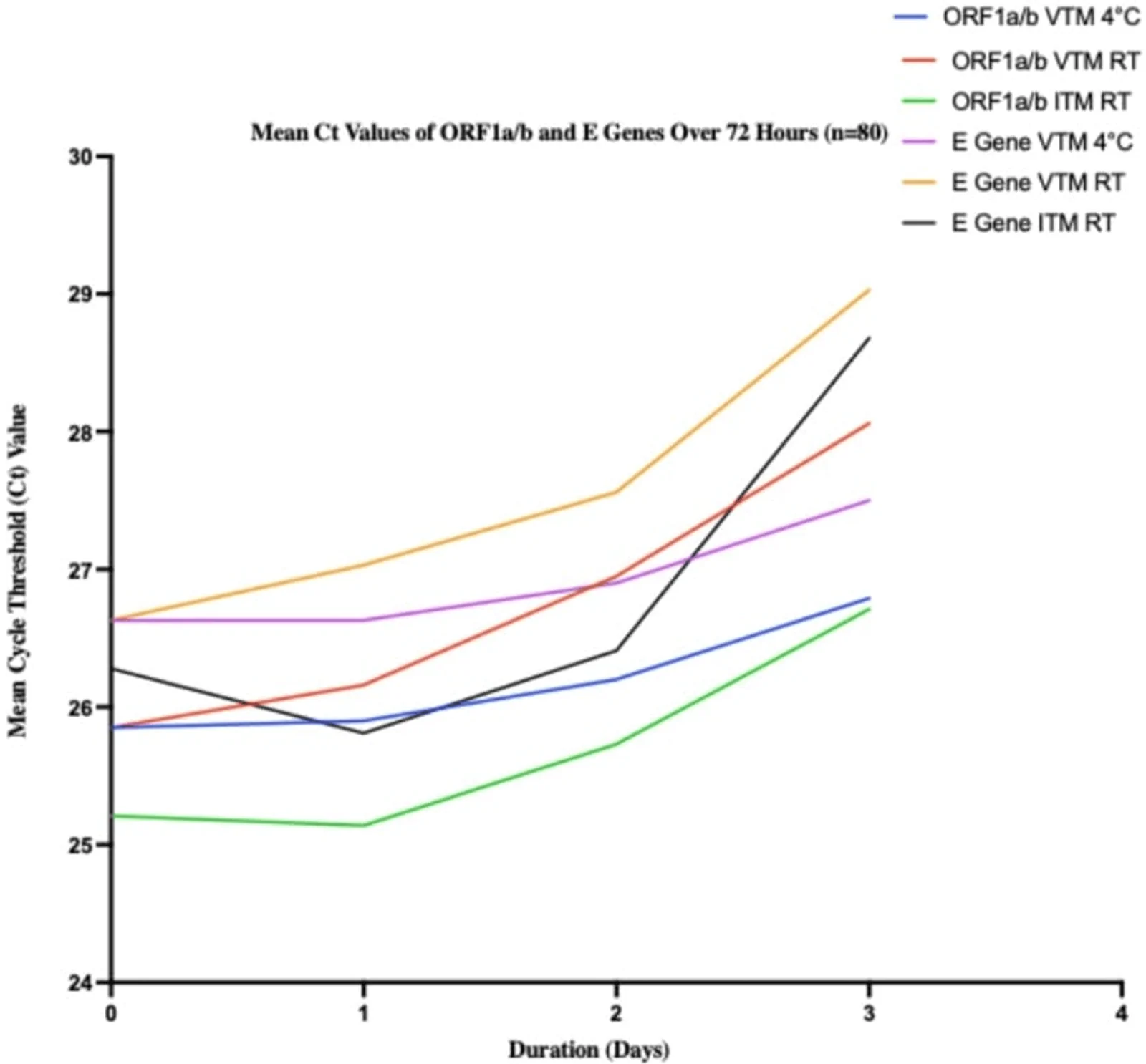
Mean Ct values of ORF1a/b and E genes in different transport media over 72 hours (N = 80) Data are presented as mean ± SD. VTM = viral transport medium; ITM = inactivation transport medium; RT = room temperature

Overall, Ct values increased with time in all transport media. The rise was greatest in VTM stored at RT. ITM stored at RT showed better stability than VTM at RT for the ORF1a/b gene, while for the E gene, ITM and VTM at RT showed similar Ct-value change at 72 hours. Refrigerated VTM showed the lowest Ct-value rise for the E gene (Figure 1).

## Discussion

The global health emergency caused by the COVID-19 pandemic officially ended on 11 May 2023 [15]. However, COVID-19 remains a global threat, and new variants of the coronavirus are still emerging. Early diagnosis and immediate treatment of COVID-19 in susceptible populations and high-risk groups is necessary to reduce the spread of the disease and break the chain of transmission. Although the CDC recommends the use of VTM or saline for the collection and transport of samples, they can present a biohazard to the healthcare workers during transport or processing in a testing laboratory. The need to maintain a cold chain and handle potentially infectious material has proven to be a major bottleneck in mass diagnostic workflows. This study aimed to evaluate ITM - a guanidine-based transport medium - and compare its performance with VTM in terms of RNA stability, safety, and diagnostic efficiency using the cobas® 8800RT-PCR system.

In the present study, samples stored in VTM at 4 °C, VTM at RT, and ITM at various temperatures exhibited stable mean Ct values for the ORF and E genes on Day 1. There was no significant difference in Ct values, indicating comparable performance in preserving viral RNA for short-term storage. At 72 hours, VTM stored at RT had a significantly higher Ct value for the ORF1a/b gene than ITM stored at RT and VTM 4 °C. For E-gene, VTM RT had a significantly higher Ct value than VTM 4 °C. This is in accordance with a study by Angoni et al., who observed an increase in the mean Ct values in samples stored in VTM at 4 °C and RT, though not statistically significant [16]. Similar to the present study, Basso et al. also reported a slight increase in Ct value within the first 48 ​hours, suggesting a potential decay of the viral molecular signal. They reported that temperature and time of storage of nasopharyngeal swabs for SARS-CoV-2 testing did not affect the reproducibility of the RT-qPCR results significantly [17]. Mosscrop et al. in their study stored SARS-CoV-2-positive samples in VTM at different temperatures of 4 °C, 20 °C, 30 °C, and 40 °C for up to five days. They found a slight increase of 0.4 Ct value at 20 °C after 24 hours, with a maximum increase in Ct value of 1.34 when held at 40 °C. They reported that samples in VTM remained stable, independent of the cold chain for up to five days [18]. This is similar to the present study, which showed a slight increase in mean Ct value on subsequent days of storage. Contrary to the present study, Yilmaz Gulec et al. in their study in samples stored in VTM at 4 °C and RT revealed that Ct values remained unchanged in the first three days, while the Ct values of samples stored at RT increased after the third day [19]. The degradation of viral RNA in VTM at RT, as indicated by higher Ct values, supports the premise that ambient storage without viral inactivation can compromise diagnostic sensitivity. This reinforces the need for either strict cold chain adherence or the use of alternative media such as ITM that allow RT stability without compromising RNA integrity.

A gradual increase in Ct values was observed over time in all media types, consistent with expected RNA degradation. This trend was more evident in VTM at RT, indicating its lower reliability for longer storage. ITM stored at RT showed better stability than VTM at RT for the ORF1a/b gene, while for the E gene, ITM and VTM RT showed similar Ct values.

These observations align closely with several prior investigations that have examined RNA preservation under different transport and storage conditions. Saini et al. in their study evaluated a guanidine-based ITM called Supra Sens SSTM and found that it inactivated the virus within 15 minutes and preserved RNA quality for up to 72 hours at ambient temperatures between 15 °C and 40 °C [20]. Banik et al. demonstrated that eNAT (a guanidinium thiocyanate-based buffer) can rapidly inactivate SARS-CoV-2 present in saliva samples and that the RNA is stable in eNAT for at least 48 hours at RT or below, even when tested at very low viral load [13]. Erster et al. found that RT-qPCR tests of patients were significantly more sensitive with nucleic acid stabilization and lysis buffer (NSLB, lysis buffer supplemented with nucleic acid stabilization mix) sampling, reaching detection threshold 2.1 ± 0.6 (mean ± SE) PCR cycles earlier than VTM samples from the same patient, and re-extracting RNA from NSLB samples after 72 hours at RT did not affect the sensitivity of detection [7]. Perumal et al. reported samples to be stable for up to 48 hours, but there was a slight decline in viral RNA quantity at day three in samples stored in lysis buffer at ambient temperatures [11]. However, Wiraswati et al. did not find significant variation in Ct values up to 18 days in samples stored in a guanidine-based inactivation transport medium at ambient temperature, as well as in samples heated for three hours at 40 °C [21]. Verma et al. in their study demonstrated that throat swabs stored in ITM retained RNA detectability more effectively than those in VTM when subjected to RT for 48-72 hours and concluded that SARS-CoV-2 remains stable for up to five days at ambient temperature and 37 °C [22].

Limitations of the study

The following are the limitations of the study. (1) This was a laboratory-based study; therefore, clinical data of patients could not be included. Ideally, samples should have been tested in triplicate to get more accurate results, but due to insufficient sample volume, it could not be done. (2) Viral culture was not performed to assess the viral inactivation of ITM. (3) Selection bias cannot be ruled out as samples were collected from a single centre. (4) Effects of higher temperatures (35-45 °C) should have been tested, as, during summer, the temperature in Rajasthan, India, goes very high.

## Conclusions

ITM can be used as a transport medium for molecular diagnostic tests for SARS-CoV-2. The use of ITM can potentially eliminate the need for cold chain logistics during sample transport, reduce the biosafety risks associated with handling infectious specimens, and improve workflow efficiency in large-scale testing setups. Moreover, as it maintains sample stability at RT (25-32 °C), it can be used across varied environmental conditions, which is particularly relevant in resource-limited testing scenarios and where samples have to be transported from remote areas. Though the study was done on SARS-CoV-2, which is no longer causing a pandemic or an epidemic, this study has relevance for using the media for transporting samples using VTM/ITM for other respiratory viruses that have high epidemic pandemic potential. Quick, safe, and reliable means of transport are required for sending samples to reference labs whenever a new virus emerges, when sample integrity, turnaround time, and biosafety are major concerns, all of which may be addressed by using ITM.
